# Ferroptosis: A critical link to treatment resistance in esophageal carcinoma

**DOI:** 10.1016/j.isci.2025.112901

**Published:** 2025-06-14

**Authors:** Ming-Xin Tang, Jin-Feng Chen, Fa-Zhi Zhao, Jun Peng

**Affiliations:** 1 Department of Gastroenterology, Liyuan Hospital of Tongji Medical College, Huazhong University of Science and Technology, Wuhan, China; 2 Department of Head and Neck Surgery, Sichuan Clinical Research Center for Cancer, Sichuan Cancer Hospital & Institute, Sichuan Cancer Center, University of Electronic Science and Technology of China, Chengdu, China; 3 Department of Gastric Surgery, Sichuan Clinical Research Center for Cancer, Sichuan Cancer Hospital & Institute, Sichuan Cancer Center, University of Electronic Science and Technology of China, Chengdu, China; 4 Department of Thoracic Surgery, Sichuan Clinical Research Center for Cancer, Sichuan Cancer Hospital & Institute, Sichuan Cancer Center, University of Electronic Science and Technology of China, Chengdu, China

**Keywords:** Biological sciences, Cancer systems biology, Health sciences, Internal medicine, Medical specialty, Medicine, Natural sciences, Oncology

## Abstract

Ferroptosis, an iron-dependent form of regulated cell death driven by lipid peroxide accumulation, induces lethal oxidative damage and disrupts cell membrane integrity. Its role in malignant tumors, such as esophageal carcinoma (EC), is increasingly recognized, offering a promising therapeutic avenue to overcome treatment resistance. Emerging evidence highlights the involvement of genes, proteins, the metabolism of metal ions, and tumor microenvironmental factors in modulating ferroptosis-associated resistance mechanisms in EC. This review systematically outlines current insights into ferroptosis in EC resistance and explores novel therapeutic strategies, including ferroptosis-targeted agents, nanotechnology, natural compounds, and multimodal approaches. Nevertheless, overcoming EC resistance remains a significant clinical challenge, warranting further investigation.

## Graphical abstract

**Figure:**
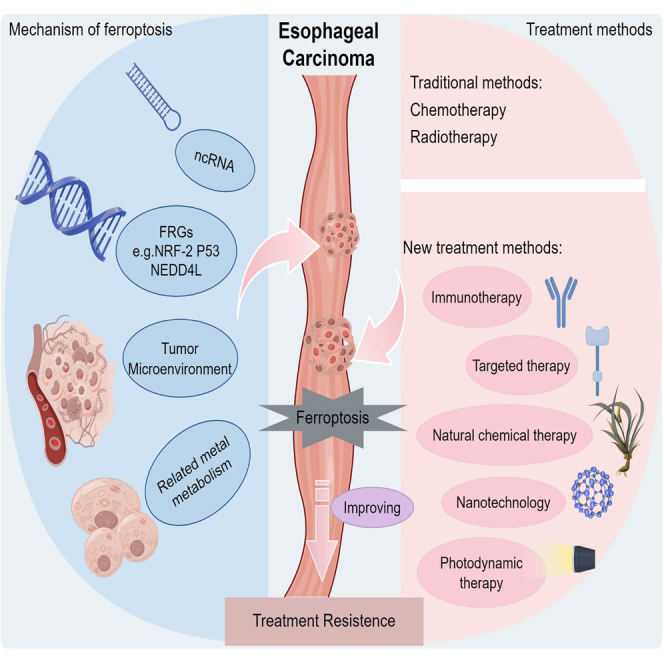

## Introduction

The incidence and mortality rates of esophageal carcinoma (EC) remain high globally. According to the GLOBOCAN 2022 estimates, EC ranks 11th in terms of incidence and 7th in terms of mortality among all cancers worldwide.^1^ With the global aging tendency, even by 2050, the number of new events of EC will increase by roughly 80.5% compared to 2022, and deaths will go up by 85.4%.^2^ Esophageal squamous cell carcinoma (ESCC) and esophageal adenocarcinoma (ECA) are the two main types of EC, and the pathological types vary in different regions. There are no discernible symptoms in the early stages of both, and symptoms only appear in the middle and late stages of progression, which often do not attract the attention of patients in time. At the same time, it is limited by diagnostic screening methods, which is already in the advanced stages when it is discovered.^3^ In terms of treatment, early-stage EC is mainly treated with endoscopic surgery, such as Endoscopic Submucosal Dissection (ESD). The main treatment for advanced EC is systemic therapy, which includes chemoradiotherapy (CRT), targeted therapy, and immunotherapy.^4^ Although these methods are constantly being improved, chemotherapy resistance is increasing year by year, and radiotherapy sensitivity is decreasing gradually, resulting in an increased risk of tumor recurrence and metastasis. The quality of patients’ survival with advanced EC is relatively poor, and the 5-year survival rate is even less than 20%.^5^ The phenomenon of treatment resistance in EC is intricate and encompasses multiple causes and mechanisms. Understanding the molecular mechanisms behind its occurrence is essential for exploring new treatment targets and response techniques to enhance the therapeutic impact and prognosis of EC.

Programmed cell death (PCD) is a necessary part of human growth and development, which regulates various life activities of the body. Traditional PCD includes autophagy, apoptosis, necroptosis, and pyroptosis. Emerging PCD includes cuproptosis, ferroptosis, PANoptosis and anoikis.^6^^,^^7^ The primary characteristic of ferroptosis, an iron-dependent PCD, is an accumulation of lipid peroxides (LPO). It is characterized morphologically by a decrease in mitochondrial cristae, shrinkage and deformation, chromatin condensation, and an increase in the density of cell membranes and vesicle membranes. It is also associated with glutathione (GSH) depletion, glutathione peroxidase 4 (GPX4) inactivation, and reactive oxygen species (ROS) accumulation.^8^ In recent years, numerous studies have demonstrated that one effective anti-tumor technique is to activate. The mechanisms in malignant tumors, such as breast, lung, and EC, have been widely studied, and corresponding drug research has moved to a new level.^9^^,^^10^^,^^11^

Drug resistance is currently the main challenge in cancer treatment. It is mainly related to DNA damage repair, the abnormal expression of oncogenes and key enzymes, hypoxia, inflammation, fibroblasts, immune cells, and the tumor microenvironment (TME) that stimulate immunosuppression. Currently, more than half of EC cancers are resistant to CRT.^12^ Although the newly added immune checkpoint inhibitors (ICIs) in recent years have had a relatively positive effect on EAC and ESCC in combination with CRT, the therapeutic effect is still limited. It is still affected by problems such as immune tolerance and target dislocation, and only 30%–40% of patients benefit from ICIs.^13^ Ferroptosis appears to be crucial to each of these procedures, which is expected to become a reliable way to break through these limitations. Therefore, this article first summarizes the mechanism of ferroptosis in the occurrence of EC and its contribution to resistance against CRT and immunotherapy. In order to further investigate the possible involvement of ferroptosis in reversing EC drug resistance, the current research development on the therapy of EC is subsequently discussed. This not only helps us to understand the drug resistance mechanism of EC in depth, but also provides the foundation for theories of novel therapies.

## Mechanisms and drug resistance of ferroptosis in esophageal carcinoma

**Figure 1:**
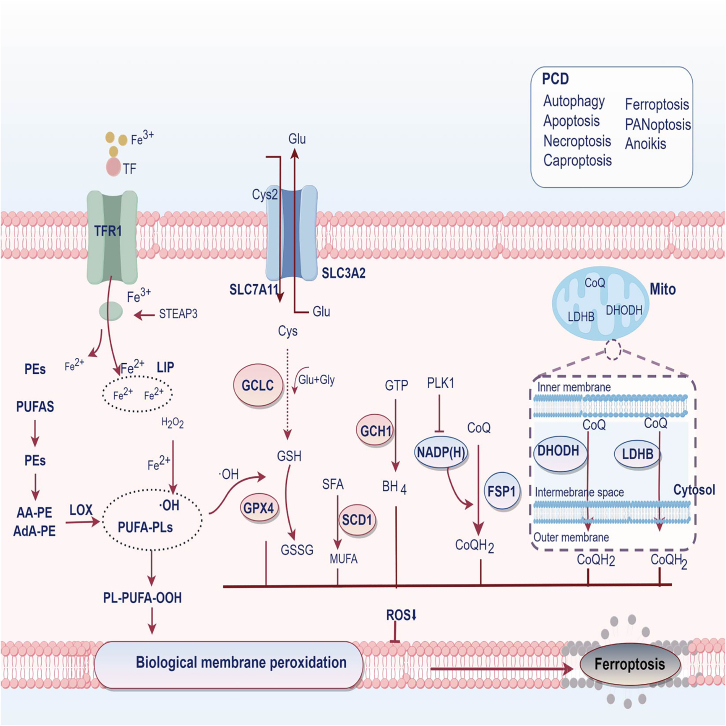
Diagram of the ferroptosis mechanism (Figdraw 2.0 was methodically employed) The major pathways of ferroptosis include both SLC7A11/GPX4 and non-GPX4-dependent pathways that lead to lipid membrane peroxidation, resulting in cellular ferroptosis. PCD, Programmed Cell Death; TF, Transferrin; TFR1, Transferrin Receptor 1; STEAP3, Six Transmembrane Epithelial Antigen of the Prostate 3; PUFAs, Polyunsaturated Fatty Acids; AA-PE, Arachidonic Acid-Phosphatidylethanolamine; LOX, Lipoxygenases; LIP, Labile Iron Pool; SLC7A11, Solute Carrier Family 7 Member 11; SLC3A2, Solute Carrier Family 3 Member 2; GCLC, Glutamate Cysteine Ligase Catalytic Subunit; SCD1, Stearoyl-CoA Desaturase 1; SFA, Saturated Fatty Acids; MUFA, Monounsaturated Fatty Acids; GCH1, GTP Cyclohydrolase 1; BH4, Tetrahydrobiopterin; CoQ, Coenzyme Q; CoQH2, Coenzyme QH2; PLK1, Polo-like Kinase 1; DHODH, Dihydroorotate Dehydrogenase; LDHB, Lactate dehydrogenase B.

The core of ferroptosis occurrence includes: cysteine starvation, GSH depletion, GPX4 inactivation, and lipid metabolism. In addition, it is also affected by TME, pH, temperature, and microorganisms. Figure 1 shows the main mechanisms of ferroptosis.

### The key pathway of ferroptosis

#### Classical pathway system Xc−

SLC7A11, also known as xCT, and SLC3A2, are important members of the solute carrier family. It is also an important component of the Cystine/Glutamate antiporter protein, which together form system Xc−. The former primarily facilitates the transport of extracellular cystine into cells. It serves as a crucial protein in ferroptosis processes.^14^ GSH is synthesized from cysteine, which is imported into the cell via SLC7A11. It can help to eliminate peroxides and maintain the intracellular oxidation-reduction homeostasis.

GPX4, a crucial enzyme in the system Xc-pathway, helps reduce LPO accumulation within cells. The Xc--GSH-GPX4 plays a crucial role in preventing ferroptosis and safeguarding cellular membranes from damage inflicted by reactive oxygen and nitrogen species, which is essential for tumor proliferation and migration.^15^ Li et al. found that the expression of SLC7A11 was significantly elevated in ESCC. When SLC7A11 was blocked, GPX4 expression levels decreased, along with ferroptosis increased.^16^ Targeted inhibition of SLC7A11 can inhibit tumor proliferation, invasion, and migration. It is an important new target for inducing ferroptosis and overcoming drug resistance in cancer therapy.^17^ Additionally, another enzyme in the Xc-system, SLC3A2, also affects ferroptosis in ESCC. Methionine (Met), which is synthesized into cysteine through the transsulfuration pathway, serves as a key substrate for GPX4 and GSH. It is found that when methionine/cystine is restricted in ECA 109, ferroptosis is stimulated through the positive feedback loop between SLC43A2 and NF-κB signaling pathways, thereby accelerating the progression of ESCC.^18^

#### Non-GXP4 dependent pathway

GXP4-independent mechanisms are also involved in the regulation of ferroptosis. GTP cyclohydrolase 1 (GCH1) is a key enzyme in the tetrahydrobiopterin (BH4) synthesis pathway. It can restore BH4 levels in cells and reduce ROS production, which is a ferroptosis mechanism independent of GXP4.^19^ In EC, the inhibition of GCH1 expression significantly suppressed cell proliferation and migration, which may also be related to this mechanism.^20^ In addition, ferroptosis suppressor protein-1 (FSP1) can resist ferritin and functions mainly in the cytoplasm. It prevents free radicals from attacking the lipid bilayer, reduces CoQ depletion and decreases ferroptosis.^21^ A Japanese research performed immunohistochemically analysis of FSP1 and GPX4 expression in surgical specimens from 97 patients with ESCC. They found that the expression of FSP1 and GPX4 was significantly increased. When the inhibitors of both were used in three ESCC cell lines, KYSE 30, KYSE 510, and KYSE 520, it was found that they could significantly induce ferroptosis in the cells.^22^

Mitochondrial ROS (mtROS) can generate lipid peroxides, which in turn induce cellular ferroptosis, so controlling the accumulation of mtROS in mitochondria is also an important way to regulate ferroptosis. Furthermore, dihydroorotate dehydrogenase (DHODH) inhibits ferroptosis by reducing CoQ to CoQH2 in mitochondria.^23^ The Marti TM team found that in mitochondria, lactate dehydrogenase B (LDHB) has a similar principle of action to this pathway, inhibiting mitochondrial ferroptosis by decreasing the ubiquinone (coenzyme Q, CoQ) to ubiquinol (CoQH2) ratio, and that when LDHB is silenced, tumor radiosurgery sensitivity of tumors was increased.^24^ In addition, mitochondrial complex I (MCI) was found to have a similar mechanism of action on mitochondrial ferroptosis.^25^ The three mechanisms, GPX4, DHODH and LDHB, all exist independently of each other. In addition, mitochondrial autophagy inhibits intracellular lipid peroxidation and ROS accumulation, thereby resisting ferroptosis.^26^ When external conditions such as nutrient deficiencies and cellular oxidative stress are affected, the mitochondria may be subjected to mutual fusion, disorders of CoQ and mitochondrial photodynamics, which can lead to a cellular susceptibility to ferroptosis and ultimately affect the cellular tumor growth process.^27^

The pathway that promotes ferroptosis and LPO accumulation is through the peroxidation of polyunsaturated fatty acids (PUFAs). First, the production of labile iron pool (LIP) can generate Fe^2+^ and H_2_O_2_, which then go through the Fenton Reaction to create the highly oxidizing hydroxyl radicals ·OH and cause ROS to accumulate. Next, the PUFA is activated by the lipoxygenase (LOX) and ultimately damages the intracellular lipid membrane, leading to ferroptosis.^28^^,^^29^ Conversely, stearoyl-CoA desaturase1 (SCD1) is a key enzyme that converts saturated fatty acids (SFAs) to monounsaturated fatty acids (MUFAs). MUFA is less susceptible to oxidation than PUFAs, resulting in lower intracellular levels of reactive oxygen species and inhibition of ferroptosis.^30^

### The regulatory gene of ferroptosis

#### Nuclear factor erythroid 2-related factor 2

NRF2 is an essential gene in the regulation of ferroptosis. Under typical conditions, NRF2 exists in an inactive state in the cytoplasm, associating with Kelch-like ECH-associated protein 1 (KEAP1). Under oxidative stress, the cell can translocate to the nucleus and dissociate from KEAP1, thereby promoting the expression of downstream ferroptosis-related genes (FRGs) that involve SLC7A11 and GPX4.^31^ The expression of NRF2 and its transcriptional targets is elevated when KEAP1 is absence, leading to the resistance of ferroptosis in squamous cell carcinoma.^32^ In both EAC and ESCC, the suppression of NRF2 expression markedly diminishes ferroptosis markers SLC7A11/GPX4 and facilitates ferroptosis, which finally enhances patients' resistance to CRT and elevates the survival rate of EC.^31^^,^^33^ Simultaneously, NRF2 facilitates the epithelial-mesenchymal transition (EMT) of esophageal ESCC cells, enabling the infiltration of numerous M2 macrophages, hence enhancing cell migration, invasion, and therapeutic resistance. This approach also enhances cellular susceptibility to ferroptosis, rendering metastatic tumor cells more vulnerable to ferroptosis inducers and demonstrating duality.^34^

#### P53

P53 is a vital tumor suppressor gene. Wild-type P53 plays a key role in regulating the cell cycle, DNA damage repair, and apoptosis. It also prevents the proliferation and carcinogenesis of damaged cells. However, P53 is one of the most frequently mutated genes in the human genome. This inhibitory role is lost when mutations occur, promoting tumor growth and compromising treatment effectiveness. Research has demonstrated that P53 can prevent SLC7A11 from producing glutathione, thereby increasing LPO and promoting ferroptosis in EC.^35^ When the body lacks or has a mutation in P53, it gradually begins to develop resistance to CRT.^36^ P53 can also be inhibited by Hsp27, a protein produced during cellular stress, leading to the downregulation of SLC7A11/GPX4 and inhibiting ferroptosis in EC stem cells.^37^ Prenetapopt (APR-246) is a P53 activator. A phase Ib/II clinical trial showed that when P53 is inhibited with APR-246, it can increase the consumption of intracellular glutathione, dramatically decrease SCL7A11 expression, and induce ferroptosis in ESCC. It has been confirmed that SCL7A11 can be used as a prognostic indicator for the treatment of patients with P53 deficiency.^38^

#### NEDD4L

NEDD4L is a member of the E3 ubiquitin ligase family, crucial for tumor growth and protein degradation. Studies have shown that this ubiquitinase is most closely associated with SLC7A11. NEDD4L is poorly expressed in ESCC, and further studies have shown that it directly binds to SLC7A11 through its WW and HECT domains. It can reduce the stability of SLC7A11 in the proteasome, promoting ferroptosis and suppressing lymph node metastasis in cancer cells.^39^ It can also inhibit cell viability by ubiquitinating c-Myc, inhibit glutamate metabolic activity, and affect ferroptosis.^40^ In addition, Chen demonstrated through *in vitro* studies that NEDD4L accelerates ferroptosis by ubiquitinating KLF5. This leads to ROS accumulation and effectively enhances the radiotherapy sensitivity of ESCC.^41^

#### Expression of other ferroptosis-related genes in esophageal carcinoma

Aberrant expression of FRGs is an important indicator of tumor growth, metastasis, invasion, overall survival, and prognosis in patients.^42^ Therefore, understanding the processes underlying their expression is essential for the prevention and treatment of EC. First, FRGs may directly affect the System Xc-pathway. High expression of cysteinyl-tRNA synthetase 1 (CARS1) in EC is usually associated with poor prognosis. Further studies have found that CARS1 may significantly reduce GPX4 expression, leading to increased ROS and malondialdehyde (MDA) accumulation, which in turn causes ferroptosis.^43^ In ECA, a predictive model composed of CARS1 and FRGs such as GCLM, GLS2 and EMC can effectively predict overall survival and prognosis.^44^ Stanniocalcin 2 (STC2) inhibits ferroptosis by activating protein arginine methyltransferase 5 (PRMT5), which increases the expression of SLC7A11, making ESCC resistant to radiotherapy. Thus, STC2 may be a new breakthrough in tumor resistance in ESCC.^45^ Another gene, Aurora kinase A (AURKA), is considered to be a pro-cancer enzyme in gastrointestinal tumors such as gastric and colorectal cancers. In investigations involving ESCC cells, the knockdown of AURKA correlates with the diminished expression of GPX4 and SLC7A11, a decrease in intracellular GSH, and the suppression of ferroptosis, as indicated by increased levels of MDA and Fe^2+^.^46^ The histone demethylase JMJD2A binds to SMARCA4 in EC, affecting chromatin structure, increasing the expression of GPX4, and inhibiting radiotherapy-induced ferroptosis. These effects increase radiotherapy tolerance and lead to immune escape.^46^ Unlike JMJD2A, TAGLN suppresses the transcription of SLC7A11/GPX4 and facilitates ferroptosis *in vivo*, mostly through its interaction with P53, and significantly contributes to the migration and invasion of ESCC.^47^

Second, FRGs regulate the ferroptosis process in cells by influencing proteins related to iron metabolism, which in turn affects the sensitivity of cells to medication. For example, SCARA5, a protein-coding gene that is expressed at low levels in ESCC, can bind to intracellular ferritin, leading to increased levels of ROS and Fe^2+^. This promotes morphological changes in mitochondria, further triggering ferroptosis and thereby inhibiting cell proliferation and migration.^48^ In contrast, ABCB7 regulates intracellular iron homeostasis through transferrin and affects intracellular LPO accumulation.^49^ Knocking down ABCB7 in EC cell lines ECA 109 and KYSE 30 increases intracellular iron ion concentration, leading to non-apoptotic cell death. ABCB7 also promotes EMT transformation through TGF-β/Smad signaling.^50^ These processes may have a special relationship with ferroptosis.

Finally, FRGs also modulate ferroptosis through specific chemical modifications. PKCiota is a common proto-oncogene, expressed in a variety of malignancies and highly expressed in EC. It can phosphorylate CDK1, accelerating the G1/S transition of cancer cells and promoting ESCC cell proliferation. It also suppressed ferroptosis in ESCC cells by inhibiting USP14-mediated GPX4 autophagic degradation.^51^ DNAJB6 can promote ferroptosis by reducing the expression of GPX4 through the phosphorylation of AKT (*p*-AKT) and inhibit the lymph node metastasis of ESCC.^52^

#### Non-coding RNAs

A growing number of studies have demonstrated that non-coding RNAs (ncRNAs), including microRNAs, long ncRNAs, and circRNAs, mediate ferroptosis pathways and contribute significantly to the development of EC. These ncRNAs have been shown not only to contribute to tumor drug resistance but also to serve as potential clinical indicators of disease prognosis or drug resistance.^53^ They regulate ferroptosis by modulating iron metabolism, GSH metabolism, NRF2 and P53-related pathways, affecting the expression of SLC7A11, LPO, and iron deposition. The three types of ncRNAs often interact to form ceRNAs that work together to regulate ferroptosis. For instance, the downregulation of lncRNA BBOX1-AS1 can upregulate miR-513a-3p, inhibiting cell growth and accelerating apoptosis and ferroptosis in ESCC.^54^ Whereas circPDE3B indirectly inhibits ferroptosis and promotes ESCC development through the HNRNPK/SLC7A11 and miR-516b-5p/CBS axis^55^; circ_000014/miR-527 reduces the sensitivity of oral squamous cell carcinoma cells to ferroptosis and increases their resistance to cisplatin (DDP) by regulating SLC7A11 expression.^56^ Lastly, the circRNA circPVT1 influences ferroptosis and the Wnt/β-catenin pathway via the miR-30a-5p/FZD axis, which causes EC to be resistant to 5-fluorouracil (5-FU).^57^ YTHDF1 is an RNA-binding protein that recognizes N6-methyladenosine (m6A) modifications. Highly expressed YTHDF1 is involved in the regulation of several ceRNA networks, such as the PAXIP1-AS1/hsa-miR-376c-3p/YTHDF1 axis, the THUMPD3-AS1/hsa-miR-655-3p/YTHDF1 axis, and the SNHG20/hsa-miR-655-3p/YTHDF1 axis.^58^ Through their influence on YTHDF1 expression, these networks control genes linked to ferroptosis and accelerate the development of EC.

In addition, ncRNAs often synergistically regulate coding genes. For example, low expression of ADAM23 and high expression of ARHGEF26-AS1 and miR-372-3p together promote ferroptosis, which inhibits cell proliferation and migration in ESCC cells. Mechanistically, ARHGEF26-AS1 upregulates ADAM23 expression by competitively binding to miR-372-3p.^59^ Ferroptosis-related proteins can also be directly bound by ncRNAs, thereby regulating ferroptosis, affecting EC proliferation and migration. EC exhibits significantly elevated SCD1 expression. Notably, miR-181a-5p can target SCD1, thereby inhibiting the proliferation, migration, and invasion of ESCC.^60^ In addition, TMEM44-AS1 (lncRNA) inhibits ferroptosis and promotes ESCC progression by binding to IGF2BP2 to increase the stability of GPX4 mRNA.^61^

**Figure 2:**
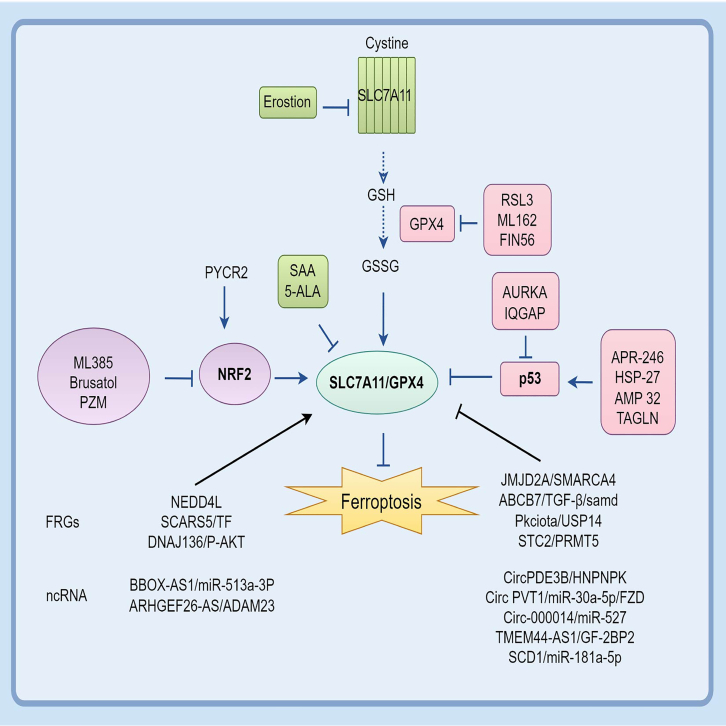
Ferroptosis-related genes pathways and corresponding targeted drugs in EC The SLC7A11/GPX4 pathway has complex network pathways, including its upstream regulators, downstream effector molecules, and potential target drugs. The most studied FRGs NRF2 and P53. FRGs, Ferroptosis-related genes; NRF2, Nuclear factor erythroid 2-related factor 2; P53, Tumor Protein p53; ncRNA, non-coding RNA; SAA, Serum Amyloid A; 5-ALA, 5-Aminolevulinic Acid.

In conclusion, ncRNAs play critical roles in regulating ferroptosis and associated signaling pathways, significantly influencing the incidence, progression, and treatment responses of EC. Figure 2 shows the network pathways of FRGs and the corresponding targeted drugs in EC.

### Ferroptosis-related metal metabolism

#### Iron metabolism

Through transferrin receptor 1 (TFR1), extracellular Fe^3+^ binds to transferrin (TF) and enters the cells, where it is converted to Fe^2+^. Some of the Fe^2+^ is stored in ferritin, while the remainder is released as free iron into the LIP of the cell, where it participates in various cellular processes. Labile iron produces a large number of hydroxyl radicals via the Fenton Reaction, which in turn promotes the lipid peroxidation of PUFAs. Ferroptosis is significantly reduced when the concentration of iron ions is lowered using iron chelating agents.^62^ Since ferroptosis depends on the accumulation of Fe^2+^, maintaining iron homeostasis is crucial for cells. EC is often associated with Fe2+ deficiency.^63^ According to a meta-analysis, a 15% lower risk of EC can be achieved by increasing daily consumption of iron by 5 mg.^64^ However, iron becomes a risk factor for EC when excessive iron intake leads to iron overload. Iron not only provides essential trace elements but also stimulates ferroptosis, demonstrating a dual role. In conclusion, preserving iron homeostasis is essential for tumor prevention and therapy to prevent iron deficiency and avoid the potential hazards of iron overload.

#### Copper metabolism and cuproptosis

The absorption and metabolism of iron and copper ions usually occur simultaneously and affect each other in regulating cell growth. Labile copper ions are involved in copper-dependent PCD, specifically cuproptosis. Unlike ferroptosis, cuproptosis depends on mitochondrial respiration. It can cause lipoylated proteins to aggregate and participate in the Fenton Reaction, leading to the generation of ROS and cellular damage. In contrast, unstable copper ions can undergo Fenton-like Reactions to generate ROS, which can damage iron-sulfur (Fe-S) cofactors, thereby affecting iron metabolism and potentially reducing the sensitivity of cells to ferroptosis.^65^ In addition, Cu^2+^ can also undergo redox reactions with GSH to ·OH through the Fenton Reaction, which consumes GSH and glucose while also inducing ferroptosis.^66^ As the Cu/Fe balance is disrupted, intracellular iron ion concentration is reduced, leading to the inhibition of ferroptosis.^67^ Some research has even proposed that ferroptosis is a sort of copper-iron-dependent cell death.^68^ Using a bioinformatics database, researchers have established a prognostic model for EC based on copper-related death and FRGs. It was observed that cells inducing both ferroptosis and cuproptosis have a comparatively extended survival duration, indicating that the concurrent activation of these pathways may offer a novel approach for the treatment of ESCC.^69^ Bai et al. used this correlation to design CuO Nanozymes, which can enhance ferroptosis-cuproptosis by accelerating the intracellular consumption of Cys and GSH and increasing the intracellular Cu^2+^ concentration.^70^ Although certain investigations have demonstrated that excessive deposits of copper ions may trigger cell death, there is currently a dearth of further research on the specific function of cuproptosis in ferroptosis of EC, notably its impact on how ferroptosis occurs.

#### Zinc metabolism

Zinc deficiency has been linked to oxidative stress, DNA damage repair and tumor apoptosis, according to earlier research.^71^ Yang’s team found in zinc-deficient ESCC that lipid peroxidation was inhibited, which may be associated with increased glycolysis and lactic acid. It leads to the inhibition of PUFA peroxidation through the lactic acid-mediated SREBP1/SCD1 pathway, resulting in ferroptosis resistance.^72^ Significantly, when the level of Zn^2+^-dependent phosphorylation was increased by inhibiting zinc transporter protein (ZIP8) expression, an increase in GPX4 expression was observed, and ferroptosis was inhibited.^73^ By constructing an animal model of zinc-deficient ESCC, it was found that correcting the zinc deficiency reduced the cancer incidence by 47%.^74^ A phase I clinical trial found that zinc gluconate treatment has the potential to prevent Barrett’s esophagus and inhibit EC. This effect is likely linked to its ability to indirectly activate tumor suppressor genes such as P53, and downregulate key miRNA expressions.^75^ This process may also indirectly promote downstream ferroptosis. Thus, correcting zinc deficiency in the body is crucial for effective tumor treatment.

### Ferroptosis and tumor microenvironment

#### Immune-associated tumor microenvironment

The TME is a complex ecosystem that includes various immune cells, cancer-associated fibroblasts (CAFs), endothelial cells, the extracellular matrix (ECM), as well as various trace elements and nutrients. Ferroptosis is essential for maintaining immunological homeostasis and immune suppression in the microenvironment.^76^ Based on various ferroptosis gene expression profiles, Zhang identified three subgroups of ESCC cell clusters (A-C) through bioinformatics analysis. Of these, cluster C is enriched with CD8^+^ cells and other immune cell infiltrates, while cluster A is relatively poor in immune cells, correlating with a worse prognosis.^77^ Similarly, in a study utilizing FRGs' prediction model for EC, it was found that the expression of DC and CD8^+^ T cells in low-risk genomes was significantly increased.^44^ Therefore, further exploration is necessary to investigate the relationship between TME and ferroptosis and establish diverse predictive models for genes and proteins. The efficacy of treatment for EC may be enhanced by inducing cell-specific ferroptosis in a targeted manner.^78^

Ferroptosis-related key proteins SLC7A11/GPX4 are abnormally expressed in tumors and affect the infiltration of immune cells and the treatment response in the TME. The current research investigates the correlation between GPX4 expression and the invasion of macrophages and myeloid dendritic cells in EC. The findings indicate a substantial positive connection between GPX4 levels and monocyte markers (CD14 and CD115) in endothelial cells. Moreover, heightened GPX4 expression correlates with a significant upregulation of M2 macrophage markers (VSIG4 and MS4A4A), indicating a possible function for ferroptosis in influencing immune cell polarization in the tumor microenvironment.^79^^,^^80^ For instance, when NRF2 is activated and binds to GCLM, there is an increase in GPX4 expression and aggregation of M2 macrophages, a process that leads to reduced cellular sensitivity to radiotherapy and exacerbates drug resistance.^34^ GCH1 exerts a multifaceted influence on cells, impacting not only regulating ferroptosis but also affecting the TME, promoting the enrichment of Treg cells, and affecting immune escape. This may represent one of the causes of low efficacy and cellular drug resistance in EC. Lu’s group of researchers found that the immune-related ferroptosis genes were observed to elevate the numbers of CD8 + T cells and Tregs, potentially linked to a T cell co-stimulatory action that enhances ferroptosis via interferon γ release by CD8 + T cells, thus leading to apoptosis.^81^ The TME has been observed to induce metabolic reprogramming and dysfunction in CD8^+^ T cells. Research has indicated that PD-1 blockade can increase the expression of Phospholipid Phosphatase (Plpp1). This has been shown to induce ferroptosis and to restore CD8^+^ T cells' anti-tumor efficacy.^82^ However, in immune cells, aberrant expression of the GPX4 gene can lead to elevated ROS levels in Treg cells, hence compromising their function and the overall immune response.^83^ Furthermore, CAFs represent a pivotal component of the TME, responsible for secreting various factors and interacting with the tumor. It can inhibit ferroptosis by secreting exosomes to inhibit intracellular ROS aggregation that leads to tumor drug resistance.^84^

However, some recent studies have pointed out that ferroptosis does not exhibit the traits of immunogenic cell death (ICD). It also does not directly trigger an immune response. Nevertheless, ferroptosis may indirectly induce an immune response in the body through specific molecular regulatory pathways after cell death, and the exact mechanisms remain unclear.^85^ It can be inferred that ferroptosis and immune metabolism are interrelated at the molecular level and together regulate the growth of EC. Associated genes can also serve as a prognostic indicator for patients with EC and provide valuable reference information for assessing the efficacy of immunotherapy.^86^

#### Ferroptosis-related proteins in tumor microenvironment

Ferroptosis in the TME is also affected by the metabolism of other components. Acetyl-CoA acetyltransferase 2 (ACAT2) is considered an inhibitory protein of ferroptosis and may lead to radiation resistance in ESCC.^87^ BACH1 is a serum autoantibody inhibitor. Studies have found that BACH1 is expressed at significantly higher levels in the group with lymph node metastasis of EC. It inhibits oleic acid synthesis through SCD1. This promotes tumor cells shifting from low to high oleic acid concentration to lymphatic fluid metastasis, inducing ferroptosis.^88^ Lipocalin 2 (NGAL/LCN2) is a protein involved in lipophilic molecular-type transmembrane transport, which significantly promotes migration and invasion in ESCC.^89^ Recent studies have found that LCN2 may increase the intracellular SLC7A11/GPX4 content and reduce the intracellular accumulation of peroxides by inhibiting ferroptosis-associated pathways such as PTGS2, NFR2.^90^ In ESCC, LCN2 was similarly found to inhibit ferroptosis, and high expression of LCN2 tends to predict lower survival rates.^91^ However, there are fewer relevant clinical studies, and the role played by LNC2 in the involvement of ferroptosis in EC drug resistance should be further explored in the future.

#### Other influencing factors in tumor microenvironment

Several studies confirm that when the temperature of the body increases, it inhibits ferroptosis in tumors and leads to chemoresistance. For pancreatic cancer, after the body temperature increases, p38 MAPK mediates resistance to gemcitabine by inhibiting ferroptosis.^92^ In hypoxic environments, the down-regulation of USP2 expression in ESCC cells facilitates ferroptosis and reduces drug resistance by influencing the stability and enhancing the degradation of NCOA4, resulting in iron ions and LPO accumulating, which further intensify ferroptosis.^93^ D-lactic acid accumulation also inhibits SLC4A7/GPX4 expression in ESCC stem cells, exacerbates intracellular ROS accumulation, and promotes ferroptosis.^94^ Finally, tumor-associated bacteria in the TME, such as *Pseudomonas aeruginosa*, produce a biofilm that secretes pyoverdine, a bacterial biofilm-secreted iron siderophore. By consuming iron, tumor cells are protected from ferroptosis, resulting in drug resistance. Therefore, anti-biofilm-*anti*-tumor therapy is also a relatively new direction of anti-tumor therapy. Yeung et al. proposed a combined treatment model involving *Streptococcus pyogenes* and antibiotic drugs, which can simultaneously induce ferroptosis and inhibit the production of pyoverdine by pseudomonas aeruginosa.^95^

## Addressing resistance in traditional esophageal carcinoma treatment

### Chemotherapy

Chemoresistance in EC is linked to numerous molecular mechanisms, including changes in protein expression such as PLK1, HMGA1, ALDH5A1, and NRF2. These molecular mechanisms work synergistically to affect the intracellular redox balance and ferroptosis, which in turn affect the sensitivity of chemotherapy drugs.^96^^,^^97^^,^^98^^,^^99^ Polo-like Kinase 1 (PLK1) is a serine/threonine protein kinase. Research has demonstrated that PLK1 can affect the levels of NADPH and GSH through the pentose phosphate pathway. When PLK1 was knocked out, the LPO rate was significantly increased after the irradiation of ESCC with radiotherapy, and the sensitivity of ferroptosis was increased in combination with paclitaxel (PTX), cisplatin, and radiotherapy.^97^ High-mobility group AT-hook 1 (HMGA1), which is closely related to tumor migration, has recently been found to assist ATF4 in activating SLC7A11 transcription, thereby maintaining intracellular redox homeostasis. This process inhibits the killing effect of ferroptosis on tumor cells. HMGA1 depletion has been shown to promote ferroptosis and restore the sensitivity of ESCC to DDP, boosting the effect of chemotherapy.^98^ Similarly, ALDH5A1 has been shown to promote EMT in ESCC. In Song’s study, it was demonstrated that after silencing ALDH5A1, a decrease in anti-ferroptosis proteins and an increase in pro-apoptotic protein (such as ACSL4) were found in DDP-treated ESCC cells in mice. It means that the resistance of DDP was reversed.^99^ The YAP inhibitor verteporfin (VP) can inhibit ESCC proliferation by inhibiting the Hipp/YAP pathway, which is an important signal transduction pathway for cell proliferation. The ferroptosis inhibitor ferrostatin-1 can reverse this metabolic process, potentially serving as a crucial mechanism for VP to overcome resistance to PTX in ESCC.^100^^,^^101^

Furthermore, targeting ferroptosis induction is a promising strategy to reverse chemoresistance in cases where resistance is linked to defects in certain key genes. In gastroesophageal adenocarcinoma, individuals with defects in homologous recombination (HR) and nucleotide excision repair (NER) show heightened sensitivity to PARP inhibitors, which is believed to be associated with ferroptosis. Prosz’s team found that in HR NER-deficient EAC after DDP treatment, single-cell RNA sequencing revealed increased expression of ferroptosis-related proteins.^102^ A preceding study corroborated the finding that the silencing of the glutaredoxin 5 (GLRX5) gene in head and neck squamous cell carcinoma elevates labile iron and ROS in cells, consequently inducing ferroptosis. This might significantly enhance the sensitivity to DDP and reverse the drug resistance of ESCC.^103^ Therefore, the selective silencing of essential proteins appears to be an important strategy for overcoming EC resistance and improving chemotherapy outcomes.

### Radiotherapy

Radiosurgery (RT) is a common therapeutic modality for EC. Nevertheless, it is currently encountering significant challenges related to resistance. First, the upregulation of System Xc- often indicates increased radioresistance and a poorer prognosis in tumors. As a result, some academics have suggested that blocking System Xc- can boost radiation sensitivity and improve the prognosis of patients.^104^ M6A modification is a common epigenetic modification of mRNA in eukaryotes. It regulates gene expression by affecting RNA metabolism, stability, splicing and translation. This modification can affect the mRNA stability of SLC7A11 and GPX4, thereby influencing cellular ferroptosis. Among them, METTL14, together with METTL3, forms the major methyltransferase complex (MTC) and helps adenine to undergo m6A modification. Studies have shown that silencing METTL3 activates ferroptosis in radio-resistant ESCC, thereby increasing sensitivity to radiotherapy (RT) and reversing radioresistance.^105^ A decrease in the levels of the lipid metabolic enzyme, ACSL4, was seen in radiotherapy-treated ESCC cells, potentially contributing to the diminished efficiency of radiotherapy. Nonetheless, mRNA stability diminished following m6A modification, leading to enhanced expression, which induced ferroptosis and heightened sensitivity to radiation.^106^ Consequently, the development of m6A modification-related drugs is a current research hotspot, seeking to find reliable drugs to reverse radiotherapy resistance.

## New treatment for esophageal carcinoma involves ferroptosis

### Targeted therapy

As the mechanism of ferroptosis gradually becomes clear, researchers are increasingly exploring its potential to minimize drug off-target effects. Studies demonstrate that inducing ferroptosis or modulating its pathways can overcome treatment resistance in multiple primary tumors. Currently, ferroptosis inducers (FINs) are mainly divided into three categories. Class I FINs (e.g., erastin、sulfasalazine) and class II FINs (e.g., RSL3 and ML162) induce ferroptosis by inhibiting System Xc- and GPX4. Class III FIN (FIN56) depletes GPX4 protein and CoQ.^29^ In addition to increasing therapeutic efficacy, this combination approach minimizes the risk of drug resistance that may develop from using a single medication. So, it can provide patients with a safer and more effective treatment plan to improve their survival and prognosis.^17^^,^^107^^,^^108^ In EC, SLC7A11 is also a central hub in the ferroptosis mechanism. Its targeted inhibition effectively induces ferroptosis, offering a promising therapeutic strategy. Sulfasalazine (SAS), which has been extensively studied, is a system Xc-inhibitor that inhibits the growth of numerous tumors. Recent studies have found that when EC cells TE-1 are treated with SAS for 48 h, their proliferation is significantly inhibited, with a 53.9% maximal inhibition rate.^109^ However, when ferrostatin-1 is added, the inhibitory effect is blocked, confirming that it can inhibit the proliferation of ESCC. According to certain research, P53-deficient EC cell can overcome their tumor chemoresistance when SLC7A11-inhibiting ferroptosis inducers are used. It has been proposed that the combined use of FINs and RT is an effective treatment for drug resistance in patients with P53 mutations.^110^ Moreover, targeted blockade of NRF2 is also an effective treatment option. In ECA, the NRF2 inhibitor Bristol, either alone or in combination with DDP, can also be used to observe increased lipid peroxidation and ferroptosis in cells.^33^ In an ESCC cell model, Pizotifen malate (PZM) targets NRF2 and can also promote ferroptosis and inhibit tumor growth by downregulating proteases such as G6PD and GPX4.^111^ ML385 is also an inhibitor of NRF2, another inhibitor that, when used in combination with RT to treat ESCC, was found to inhibit the RT-induced cytoplasmic-nuclear translocation of NRF2, inhibit SLC7A11, and simultaneously cause cells to enter the G2/M phase, enhancing RT sensitivity.^112^ MF-438, by targeting and inhibiting SCD1, it can effectively increase ferroptosis and ICD in ESCC and increase radiotherapy sensitivity.^113^ Finally, 5-aminolevulinic acid (5-ALA) is a naturally occurring amino acid, but because of its photosensitivity, it is often used in combination with photodynamic therapy for skin diseases or bladder cancer.^114^ However, it can also be used to induce ferroptosis in ESCC through suppressing GPX4.^115^ Despite numerous studies indicating that various ferroptosis inducers had considerable anti-tumor efficacy in cancer treatment, their translation into clinical practice encounters numerous obstacles. At the present, anti-EC methods aimed at ferroptosis are transitioning from preclinical investigations to initial clinical trials. Sorafenib, known for its ferroptosis-inducing properties, has been demonstrated to trigger ferroptosis by specifically inhibiting the System Xc-pathway. Furthermore, a previous clinical trial (NCT00917462) validated the treatment efficacy of sorafenib in individuals with refractory EC, which, in addition to its original chemotherapeutic effect, may also be associated with ferroptosis.^116^ Anticipating the future, it is projected that the organic integration of ferroptosis-inducing agents with established therapeutic modalities, including conventional chemotherapy, radiotherapy, or immunotherapy, will enhance the clinical applicability of ferroptosis-targeted therapies and expedite their clinical translation process.

### Natural chemical ingredients

Many natural chemical components derived from plants and Chinese herbal medicines have the capacity to induce ferroptosis. They trigger ferroptosis in EC by regulating iron metabolism, enhancing oxidative damage, or interfering with the cell’s antioxidant defense system.^104^^,^^117^ Broadly speaking, they are classified into two different categories: those that inhibit system Xc- and directly induce ferroptosis by increasing cellular oxidative stress, and those that inhibit ferroptosis indirectly by promoting the regulatory proteins or genes in the ferroptosis pathway. The former mainly includes: Ferulic acid and Oridonin. Ferulic acid is a phenolic substance that has the effect of scavenging intracellular ROS, as well as anti-inflammatory, antioxidant, and anti-tumor effects. Cao revealed that ferulic acid can inhibit angiogenesis in ESCC by suppressing VEGFA and PDGFB expression. It also inhibits SLC7A11 activity, induces ferroptosis, and suppresses migration and invasion.^118^ A similar natural tetracyclic diterpene, oridonin (Ori), can bind to cysteine to inhibit the gamma-glutamyl cycle, cause an increase in Fe^2+^, MDA, and ROS in the TE1 cell, which can cause ferroptosis and inhibit EC growth.^119^ Berbamine (BBM, a natural bis-benzylisoquinoline alkaloid) inhibits tumor growth, EMT and angiogenesis by inhibiting the deubiquitinase USP51, promoting the proteasomal degradation of GPX4, which also promotes ferroptosis.^120^ Additionally, the mycotoxin gliotoxin has immunosuppressive properties. It induces apoptosis, generates an abundance of ROS, and suppresses the action of the proteasome and immune cells. Studies have shown that gliotoxin inhibits SLC7A11 and GPX4, promoting ferroptosis, which is an effective natural therapy for EC.^121^

Natural compounds that affect ferroptosis by regulating FRGs include Isoalantolactone, Licochalcone A, among others.^122^^,^^123^^,^^124^^,^^125^ Isoalantolactone can induce apoptosis through the overexpression of death receptor 5 (DR5). Meanwhile, it also blocks microRNA-21-mediated PCD, leading to increased ROS accumulation and promoting ferroptosis. Isoalantolactone treatment was shown to significantly inhibit the growth of CEA109 cells in a mouse model in the Netherlands.^122^^,^^123^ Likewise, licochalcone A (LCA) acts on EC cells by upregulating P53 expression and inducing cells to enter the G2/M phase. It also significantly reduces mitochondrial membrane potential, increases membrane lipid peroxidation.^124^ Realgarly, an arsenic-containing sulfide, has been shown in several studies to inhibit tumor growth and promote apoptosis.^125^ In EC, it induces ferroptosis through the p62-Keap1-NRF2 and ROS-ASK1-p38 MAPK pathways, inhibiting the proliferation, migration, and invasion of EC cells, thereby suppressing tumor progression.^126^^,^^127^ Similarly, brusatol (Bru), a natural product extracted from Brucea javanica, functions as an NRF2-specific inhibitor. Zhu confirmed that it downregulates GCLC, SLC7A11, and FTH1, leading to the inhibition of GSH synthesis and induction of ferroptosis in ESCC. When used in combination with chemotherapeutic drugs such as DDP, it can significantly increase the sensitivity of tumor cells to chemotherapeutic drugs.^128^ All of them are listed in Table 1.

**Table 1:** Mechanisms of natural chemical ingredients

Natural compound	Chemical reaction	Results	References
Nobiletin	Zn2+↓CREB↓→GPX4↓	ferroptosis↑	Yang et al.^73^
Ferulic Acid	VEGFA ↓PDGFB↓SLC7A11↓	ferroptosis↑ Angiogenesis and migration invasion↓	Cao et al.^118^
Oridonin	GPX4↓ gamma-glutamyl cycle↓	Fe^2+^, MDA, ROS↑ ferroptosis↑	Zhang et al.^119^
Berbamine	USP51↓GPX4↓(Accelerated degradation)	Fe^2+^, ROS↑ EMT, Angiogenesis↓	Peng et al.^120^
Gliotoxin	SUV39H1↓→SLC7A11/GPX4↓	ferroptosis↑	Zhang et a.^121^
Isoalantolactone	microRNA-21↓	ROS↑→ferroptosis↑	Wen et al.^123^
Licochalcone A	P53↑→SLC7A11/GPX4↓	Keeps cells at G2/M ROS↑ →ferroptosis↑	Liu et al.^124^
Realgar	p62-Keap1-NRF2↓ ROS-ASK1-p38-MAPK↑	ROS↑→ferroptosis↑	Yang et al.^126^; Zhang et al.^127^
Brusatol	NRF2↓→SLC7A11/GPX4↓	GSH↓ROS↑→ferroptosis↑ efficacy of DDP↑	Zhu et al.^128^

CREB, cAMP-response element binding protein; GPX4, Glutathione Peroxidase 4; SLC7A11, Solute Carrier Family 7 Member 11; VEGFA, Vascular Endothelial Growth Factor A; PDGFB, Platelet-Derived Growth Factor Subunit B; MDA, Malondialdehyde; ROS, Reactive Oxygen Species; NRF2, Nuclear Factor Erythroid 2-Related Factor 2; AMPK, AMP-Activated Protein Kinase; DDP, Cisplatin.

### Photodynamic therapy

Photodynamic therapy (PDT) offers numerous benefits, including selectivity, non-invasiveness, and the ability to preserve organ structure and function as much as possible in tumor treatment. PDT can induce various types of cell death, including apoptosis, pyroptosis, ferroptosis, and necrosis. The nature of cell death predominantly relies on the photosensitizer employed.^129^ In the ESCC, using talaporfin as a photosensitizer was found to effectively increase the photodynamic effect through both direct lipid peroxidation by the generated ROS and inhibition of system Xc^−^.^130^ Aminolevulinic acid-based photodynamic therapy (ALA-PDT) can induce ferroptosis, diminish serum HMGB1 levels, and elevate LPO levels. Meanwhile, morphological alterations were also noted in the macrophage mechanism.^131^ In addition, the photosensitizer ALA can also cause a decrease in GPX4 and an accumulation of ROS in the ESCC, inducing ferroptosis by itself.^115^

### Immunotherapy

There are several characteristics of the internal immunogenic reaction associated with ferroptosis that significantly affect immunological tolerance. TME in ESCC has been found to cause immune tolerance through the suppression of various immune cells. Therefore, relieving the immune suppression within the TME is an important way to overcome ESCC tolerance.^132^ Yuan’s team found that NQO1 can activate downstream NRF2 to inhibit ferroptosis. In KEAP1-deficient head and neck squamous cell carcinoma, blocking NQO1 can induce ferroptosis while increasing the sensitivity of immunotherapy and improving immune tolerance.^32^ In another study, ferroptosis was found to play a “firing” role in the process, which can significantly increase the efficacy of immunotherapy in experiments with lung cancer cells using CAR T therapy.^133^ In addition, it is possible to improve the therapeutic resistance of TME by regulating the immune response and inducing apoptosis. For example, Chu et al. designed nanospheres that can induce apoptosis by increasing the intracellular Ca^2+^ concentration and a significant buildup of ROS, thereby causing ferroptosis and apoptosis. These injuries ultimately cause ICD, which greatly increases the efficacy of tumor therapy by modulating the immune response.^134^ The novel nanozyme Fe-MOF/CP consumes body cholesterol, increases intracellular ROS accumulation, and induces an immunogenic response in the body. It can enhance antitumor immunity when used in combination with a PD-1 blocker. This achieves a combination of ferroptosis therapy and immunogenic therapy, and reflects powerful antitumor activity.^135^ The 2DG@FS-Nb nanozyme system employs NIR-II fluorescence/photoacoustic imaging to identify tumours precisely, deplete GSH, provoke significant LPO and ferroptosis, and elicit robust ICD. It can also impede lactate generation, suppress the proliferation of M2 macrophages and diminish Treg levels, so modulating the TME and enhancing the advantages of ICD.^136^

### Special nanotechnology applications

Based on the basic principles of iron metabolism and the necessary metabolic processes, nanozymes, including precious metal-based, iron-based, magnetic nanozymes, and other nanozymes, are currently utilized in ferroptosis-based tumor therapy. However, inducing strong ferroptosis to treat cancer has been proved difficult since iron is stored in significant quantities in heme proteins (such as hemoglobin and myoglobin), and iron reserves (such as ferritin and transferrin). The key to inducing ferroptosis lies in enhancing the delivery of exogenous iron to cancer cells and facilitating the continuous conversion of Fe^3+^ to Fe^2+^.^134^^,^^137^ Fortunately, Yang’s mesoporous iron sesquioxide nanoparticle (HMISN) has been shown to catalyze the rapid cycling of Fe^3+^/Fe^2+^, achieving this goal.^138^ Some of these nanozymes induce ferroptosis by directly consuming GSH, decreasing GPX4 activity, and increasing H_2_O_2_, ultimately achieving better tumor treatment effects.^139^^,^^140^ Others increase the intracellular levels of special substances such as NO and H_2_S, which destroy critical chemical bonds and cause the indirect depletion of intracellular GSH.^141^^,^^142^^,^^143^

The combination of nanomaterials and biochemical agents can significantly improve drug delivery efficiency, increase bioavailability, and enhance the efficacy of targeted therapy. This strategy represents an effective path toward personalized treatment and has been widely investigated.^144^^,^^145^ For instance, the synthesis of an organic framework COF containing I can increase radiation-induced ferroptosis and thus increase the sensitivity to radiotherapy. This approach dramatically improved the radiotherapy resistance of EC cells.^146^ Montesdeoc’s team first reported Co (III) polypyridine sulfasalazine complex as a ferroptosis inducer, which is a supplement to metal chelates in inducing ferroptosis.^147^ Some people have assembled ferrocene methanol (FC) and oxaliplatin prodrug [OXA(IV)] into nanoparticles. FC is used to disrupt the intracellular redox balance and increase H_2_O_2_ *in vivo*. This not only accelerates ferroptosis but also significantly enhances the anticancer effect of OXA while reducing the cytotoxicity of platinum drugs.^148^

Moreover, nanotechnology enables the effective delivery of ferroptosis-related bioenzymes, proprietary medications, radiopharmaceuticals, and photoconductive materials to malignant cells. This delivery technique markedly diminishes medication toxicity while substantially enhancing bioavailability. The nanocarriers enhance medicines' targeting and tissue penetration abilities, hence advancing ferroptosis-targeted therapy significantly.^149^^,^^150^^,^^151^^,^^152^ The nanozyme with NADPH oxidase (NOX)-like activity (a biological orthogonal nanoenzymes) can disrupt the NADPH homeostasis when irradiated with NIR-II light. By depleting NADPH, it can stimulate ferroptosis and increase the efficacy of mild photothermal therapy.^149^ Semiconducting polymer Nano prodrug (SPNpro), which is also synthesized using nanotechnology, can be used to induce singlet oxygen (^1^O_2_) production using the powerful tissue penetration of US, thereby exerting the precise therapeutic effect of tumor ferroptosis. This has greater penetration than PDT and also has the characteristics of high prodrug selectivity and low toxicity.^150^ Similarly, when I^131^ and aPD-L1 nanomaterials are combined and delivered to tumors, ferroptosis is induced, ICD is amplified.^152^

Despite numerous advancements in nanomedicine research at the laboratory level, there is still insufficient evidence regarding its efficacy and safety in clinical applications. Therefore, to translate nanomedicine from laboratory research to clinical applications, more prospective studies and large-scale clinical trials are needed to verify its metabolic processes, potential toxicity, and long-term safety and efficacy.

### Multimodal combination

Combined with thermotherapy (TT), PDT, CRT, ICD, and ferroptosis, it can greatly increase the therapeutic effect and reduce toxic side effects. In order to overcome the drug resistance of EC, researchers have designed a triple-drug approach targeting GSH depletion, ferroptosis induction, and ICD. The triple-drug strategy is designed to induce SLC7A11/GPX4 downregulation and LPO accumulation, leading to mitochondrial depolarization, which ultimately increases the tumor inhibition rate and overcomes drug resistance.^153^ Employing comparable technology, Chen’s team effectively achieved a triple synergistic treatment incorporating chemotherapy, photothermal, and targeted ferroptosis, significantly enhancing the lethality against EC cells.^154^ Wang et al. studied EFP@MN, a metal-polyphenol Nano-capsule. It combines ferroptosis, PDT and αPD-L1 to enhance multimodal therapy. This strategy induces macrophage autophagy and LPO.^155^ Some nanomaterials have also incorporated PDT into traditional CRT and immunotherapy regimens to implement triple or even multi-modality therapy, providing new ideas for drug development in EC. Chen’s team combined the photosensitizer Ce6 with glucose oxidase and to produce Ce6@HGMOF nanoparticles, which use PDT to consume glucose in cells, produce H_2_O_2_, and improve the hypoxic state in the internal environment. Simultaneously, the system consumes GSH and enhances ROS accumulation, as verified in ESCC. This method ingeniously combines PDT, starvation therapy, and ferroptosis to reverse hypoxia in the TME and further enhance ferroptosis.^156^ The incorporation of the photosensitizer SR 780 produced an equivalent outcome.^157^ Li added bevacizumab, an anti-angiogenic monoclonal antibody, and combined it with PDT to create a positive feedback loop for ROS storms, accelerating EC death.^158^ Table 2 demonstrates the action characteristics of various nanocombination drugs on tumor cells.

**Table 2:** The action characteristics of nanocombination drugs

Nanomaterials	Cell Line	Characteristic	Function Principle	References
MitCuOHA Nanozymes	MCF-7	Depletion of Cys and GSH	Ferroptosis-cuproptosis	Bai et al.^70^
CaO2@CDs-Fe(CCF)	4T1 NIH 3T3	Ca^2+^, Fe^2+^	Reducing GXP4 expression and reversing TME resistance	Chu et al.^134^
2DG@FS-Nb	4 T1	D-A-D type NIR-II molecules 2-deoxy-D-glucose (2DG), PD-L1 nanobody (Nb)	Accelerated ferroptosis, amplified immunotherapy	Dai et al.^136^
CDDP@GP@HMISN@LAC	4T1 MC38	Hollow mesoporous iron sesquioxide nanoparticle	Enables rapid transformation of Fe^2+^/Fe^3+^ and generation of hydroxyl radicals. Increases the efficacy of ferroptosis	Yang et al.^138^
IB@ Fe-ZIF 8@PDFA	4T1 A549	Endogenous replenishment of consumed H_2_O_2_	Enhancing photothermal tolerance Improving the TME	Qin et al.^140^
IS@ATF	NIH 3T3	NO donor L-arginine (L-Arg)	Depletion of GSH by NO reduces GXP4 activity.	Wang et al.^141^
DSF@HMCIS-PEG-FA	4 T1	H_2_S, Cu^2+^, Fe^2+^	Accelerating cycle of ferroptosis-cuproptosis	Huang et al.^142^
Fc-siRNA@O-LNPs	MCF-7	Targeted siRNA for SLC7A11/NRF2	Enhanced ferroptosis while maintaining security	Liu et al.^145^
TADI-COF-Fc	KYSE150	I and Fe^2+^	Increases intracellular antioxidant content and promotes ferroptosis	Zhou et al.^146^
^131^I-aPD-L1	4 T1 NIH-3 T3	Gathering of^131^I and aPD-L1	Combined radiotherapy and immunotherapy	Shen et al.^152^
SANTA FE OXA	A549 Triple-MDA-MB-231	Haluronic acid oxaliplatin prodrug and ethylene glycol-coupled linoleic acid (EG-LA).	Enhancing ferroptosis and attenuating cytotoxicity of platinum drugs	Shi et al.^148^
Bioorthogonal Nanozyme	MCF-7	Contains NADPH oxidase (NOX)-like activity and glucose-6-phosphate dehydrogenase	Disruption of NADPH homeostasis, promotes ferroptosis and mild photothermal therapy	Huang et al.^149^
SPNpro	4T1	Generation of singlet oxygen	Combined acoustic dynamics and ferroptosis	Wang et al.^150^
SLNART	KYSE150 KYSE30	Artesunate, Solid lipid nanoparticle	Natural compounds combine with ferroptosis, reverse cellular resistance	Xia et al.^151^
Fe_3_O_4_@PDA-HCPT NPs	EC1 EC109	Fe_3_O_4_, polydopamine a, 10-hydroxycamptothecin	Combination of chemotherapy, photothermal and ferroptosis	Chen et al.^154^
EFP@ MN	B16	Metal polyphenol network	Immunotherapy, PDT and ferroptosis	Wang et al.^155^
Ce6@HGMOF	EC 109	Hydrogen peroxidase-like catalytic activity produces oxygen	Combination of PDT, starvation therapy, ferroptosis	Chen et al.^156^
REV@SR780Fe@LEV-RS 17	4T1	Contains SR 780 Photosensitiser and Fe^3+^.	Combination of PDT, ICD and targeted ferroptosis	Guo et al.^157^
Bev-IR 820@FeIII TA	KYSE 150	Addition of anti-angiogenic agent (Bevacizumab)	Combination of anti-angiogenesis, PDT and ferroptosis	Li et al.^158^

MCF-7 (breast cancer cell line); 4T1 (breast cancer cell line); MC38 (colonrectal cancer cell line); A549 (non-small cell Lung Cancer cell line); NIH 3T3 (embryonic fibroblast cells line); KYSE 150 and KYSE30 (esophageal cancer cell line); Triple-MDA-MB-231 (human negative breast cancer cell line); EC1 and EC 109 (esophageal cancer cell line); B16 (mice melanoma cell line). GSH, Glutathione; GPX4, Glutathione Peroxidase 4; PD-L1, Programmed Death-Ligand 1; NO, Nitric Oxide; NADPH, Nicotinamide Adenine Dinucleotide Phosphate; PDT, Photodynamic therapy.

## Conclusions and prospects

In advanced EC, the activation of ferroptosis is significant for reversing a tumor’s resistance to immunotherapy, chemotherapy, and radiation. More and more studies have also found that Chinese herbal medicine components have a targeted blocking effect on ferroptosis in EC. Therefore, targeted therapy with natural compounds against oxidative stress should be emphasized. Additionally, traditional treatments themselves serve as the first attack against tumor cells, and it is vital to establish strategies to harness the body’s immune attack capabilities through immune cells to form a "second blow". Although ferroptosis can enhance the effectiveness of immunotherapy, further research is required to determine whether it can induce ICD and maintain immune status, or whether it might lead to immune resistance.^88^^,^^159^ Another ongoing focus is how to improve targeting accuracy by causing ferroptosis in malignant cells while sparing immune cells, such as neutrophils and T cells in the TME.^17^ Last but not least, ferroptosis-related genes, in combination with various PCD methods, can be used to establish a prognostic model for the management and treatment of EC. The integration of nanotechnology to enhance the therapeutic response rate of EC by combining multiple treatment modalities is also important. However, most of the research is still at the *in vitro* or animal testing stage, lacking sufficient clinical evidence. All of these represent significant avenues for future research to translate the use of ferroptosis in EC treatment from the laboratory to clinical practice.

## Acknowledgments

The figure was created with Figdraw 2.0. The authors disclosed receipt of the following financial support for the research, authorship, and publication of this article: This study was financially supported by grants from the Sichuan Province Clinical Key Specialty Construction Project.

## Author contributions

Ming-Xin Tang: responsible for collecting, organizing, classifying literature and writing the first drafts. Jin-Feng Chen: Participate in literature analysis, assist in writing the first draft, and generated all figures. Fa-Zhi Zhao: Provide research ideas, review and revise articles. Jun Peng: Guided literature screening and analysis, reviewed, and revised the article.

## Declaration of interests

The authors declare that the article was conducted in the absence of any commercial or financial relationships that could be construed as a potential conflict of interest.

## Declaration of generative AI and AI-assisted technologies in the writing process

During the preparation of this work the authors used ChatGPT in order to improve the readability and language of the article. After using this tool, the authors reviewed and edited the content as needed and take full responsibility for the content of the published article.
